# Supplementary material for: Dismantling colonial legacies: Decolonising research and teaching at the Health in Humanitarian Crises Centre, London school of hygiene and tropical medicine
Source: PLOS Glob Public Health. 2025 Jul 23;5(7):e0004833. doi: 10.1371/journal.pgph.0004833 (PMC12286357; doi:10.1371/journal.pgph.0004833)
Supplement: Attachment — Submitted filename: Feeback log.docx [file pgph.0004833.s002.docx]

PGPH-D-25-00256

Dismantling colonial legacies: Decolonising research and teaching at the Health in Humanitarian Crises Centre, London School of Hygiene and Tropical Medicine

PLOS Global Public Health

Addressing feedback from reviewers

| **Feedback** | **How we addressed it** |
| --- | --- |
| Please address all the comments by both reviewers carefully. Have clear definitions with citations of key terms used (e.g., global health, humanitarian health). Strengthen the clarity and details in the methods section as per reviewer comments. | Thank you for the constructive feedback. Following the reviewer’s advice, we decided to focus mainly on humanitarian health and center our analysis on it to avoid confusing it with global health. |
| A rebuttal letter that responds to each point raised by the editor and reviewer(s). You should upload this letter as a separate file labeled 'Response to Reviewers'.  A marked-up copy of your manuscript that highlights changes made to the original version. You should upload this as a separate file labeled 'Revised Manuscript with Track Changes'.  An unmarked version of your revised paper without tracked changes. You should upload this as a separate file labeled 'Manuscript'.  Guidelines for resubmitting your figure files are available below the reviewer comments at the end of this letter. | Thank you. We have drafted a rebuttal letter that includes a feedback log that describes how each of the reviewers and editor points were addressed.  We clearly marked the two documents as per the instructions; manuscripts and revised manuscript with track changes. |
| Please review your reference list to ensure that it is complete and correct. If you have cited papers that have been retracted, please include the rationale for doing so in the manuscript text, or remove these references and replace them with relevant current references. Any changes to the reference list should be mentioned in the rebuttal letter that accompanies your revised manuscript. If you need to cite a retracted article, indicate the article’s retracted status in the References list and also include a citation and full reference for the retraction notice. | Thank you. We observed an error in the referencing style that did not include the DOI, which is not changed.  We added 4 additional references to the study methods, to support our selection of methods and approaches, as per the reviewers’ comments. |
| **Reviewer 1** | |
| Thank you for the opportunity to review this article. This research is an important contribution to how Global North institutions have been and should be conceptualizing and enacting decolonization in the fields of global and humanitarian health research. The findings from this research will be helpful to inform future action as to how institutions can support the decolonization agenda in humanitarian health research and how they can address limitations in doing so. | Thank you so much for the thoughtful feedback and encouraging comments. |
| **Abstract** | |
| - Clearly specify who is being referred to within the humanitarian health community—whether the discussion includes researchers, academics, practitioners, policymakers, or other groups. | Thank you for this comment. We clarified what we mean by humanitarian health communities on p. 1, and the new wording is “We conducted a qualitative study which aimed to explore the perceptions of members of the Health in Humanitarian Crisis Centre (HHCC) —including researchers and educators— and their external partners —including humanitarian health researchers, practitioners, and donors— regarding how to decolonise research, teaching and partnerships at a leading global health Higher Education institution in the UK.” |
| - Clarify the context in which the decolonization discourse is being discussed. At times, it is unclear whether the authors are referring to decolonization in academic spaces, fieldwork, or both. Providing explicit distinctions will enhance the reader’s understanding. | Thank you. We clarified the study’s space, mainly in research and teaching in institutions in the global north on p. 2. The new wording is “Our study provides the foundations for humanitarian health researchers and educators based in the Global North to begin to practically decolonise their work in the sphere of global/humanitarian health.” |
| **Introduction** | |
| -The authors appear to use "global health" and "humanitarian health" interchangeably. However, these sectors have distinct origins and mandates, and the decolonization discourse has evolved differently in each. In the first paragraph, the authors introduce decolonization in global health while referencing both sectors, but later shift focus exclusively to global health. To improve clarity, the authors could explicitly acknowledge and differentiate between both sectors, ensuring clarity when discussing the decolonization discourse in each. Currently, the text moves between them without clear distinctions. | Thanks for the comment. We received a similar comment from the second reviewer that suggested deleting the section on decolonizing global health. We followed their suggestion and delete the section on decolonising global health, and replaced that by a brief introduction on decolonising global health, followed by the definition by Aloudat’s, ‘Decolonisation Efforts at LSHTM’, then ‘Gaps and Divergent Conceptions, as you suggested on p. 6 |
| - When discussing white saviorism, it would be valuable to clarify that this concept is not solely tied to white identity. Rather, it refers to a broader mindset of individuals and groups who hold power thinking are "saving" those in vulnerable positions—one that can be perpetuated by anyone in a position of power, regardless of race or ethnicity. Acknowledging this nuance would help expand accountability, including for local elites who may also reinforce white savior dynamics in humanitarian health contexts. | Thank you for this point. Indeed, our understanding of white saviorism extends beyond racialized identity to systems of power – we have now clarified the text on p.4 to recognize this more clearly. We built on the work of Yu et al. (Yu, C. (2021). Rising Scholar: An Examination of the Institutionally Oppressive White Savior Complex in Uganda Through Western Documentaries. International Social Science Review, 97(2), 1–28.), and added the following text” While this concept originates in colonial histories that constructed “whiteness” as a racialised identity tied to moral and civilisational superiority, it is increasingly recognised not as an expression of racial identity alone, but as a broader power-laden orientation[31]. This mindset—rooted in hierarchical relations of power and benevolence—can be adopted by anyone occupying a position of privilege or institutional authority, including local elites, who may also perpetuate saviour dynamics within humanitarian responses[31].” |
| - Localization - could you briefly explain more how localization is claimed to perpetuate the existing power dynamics (p.7) | Thank you. We have expanded on the criticism of localisation building on the three most common arguments in the literature on p. 7. We added the following “International humanitarians still control funding and decision-making, limiting local actors to sub-contractual roles rather than equal partnerships, and reinforcing colonial legacies through externally defined standards and accountability frameworks [43, 44] Moreover, the influence of neoliberalism within the humanitarian system continues to pose a significant barrier to a more emancipatory and transformative agenda, and offers a predominantly technical solution to what is fundamentally a deeply political and contested issue of power and authority [44].” |
| **Methods** | |
| - Could the authors specify whether the results of the scoping review also integrated within this article, or how they have informed and influenced the development of this paper? In other words, please explain the role the scoping review had prior or during the development of this qualitative study. | Thank you for the comment. Indeed, the scoping reviewed supported the development of our study’s topic guide and codebook. We have clarified this on p. 9: “The scoping review early findings contributed to informing the development of the interview guide—particularly the probing questions—and guiding the initial analytical framework used to construct the codebook.” |
| - Could the authors clarify whether the term "donors" refers to those funding academic institutions, humanitarian organizations, or both? A brief explanation would help distinguish the different roles and influences of donors in these spaces | Thank you. In this study, we interviewed mainly donors/funders of humanitarian health research. We added these details to the text on p. 9. |
| To avoid confusion on table 1, I may suggest to change the wording to mixed race and add in parenthesis (Asian and White) | Thank you. All the participants self-defined their racial/ethnic background, occupation, grade of seniority and gender. The participant who self-identified as Mixed Asian, did not identify further racial subcategories.  We have made a few changes for clarity on p. 9. |
| Could the authors add some reference to their participatory research approach and add more details as to what this entailed throughout the research process? | Thank you for the comment. We added the following on p. 10. “The study incorporated participatory elements, drawing on principles of participatory research to foreground the lived experiences and perspectives of HHCC members and partners[34]. While not fully co-produced, the research involved collaborative member engagement in FGDs, and co-design of the charter, thereby seeking to reduce extractive dynamics and centre community-defined knowledge[35].” |
| Could the authors provide more details on the 'Data Collection and Analysis' section in the Methods. Particularly, did the authors use any methodology to conduct the qualitative study? How did the inductive coding lead to the selected themes, and was this conducted by 1 or 2 researchers? What actions did it involve to code inductively? What frameworks or research questions were used to conduct the deductive coding? | Thank you so much. We made changes to respond to your comments as follows “The qualitative study was informed by participatory elements, prioritising the lived experiences and knowledge systems of participants, and seeking to critically examine how colonial histories and power structures shape the decolonisation of research, teaching, and partnerships. Data were generated through interviews conducted online using Zoom, with 17 held in English and 3 in Arabic. All interviews were audio-recorded, and those in Arabic were translated into English to ensure consistency in the analysis. Following transcription by SH, the transcripts were inductively and deductively coded using MAXQDA software. Inductive coding involved a close reading of the data to identify patterns, meanings, and concepts emerging directly from participants' accounts. This process involved line-by-line coding, constant comparison, and iterative refinement of codes. Deductive coding was informed by sensitizing concepts derived from the scoping review and the overarching research questions. Themes were subsequently developed through a process of synthesis and interpretation of the coded data by SH, with regular discussions among the research team to validate findings. While we intended to develop race-related themes, as suggested in the literature [2, 14], participants ultimately did not focus extensively on this.” |
| **Results** | |
| Would it be possible to add a quotation or example of this claim on pp. 12: “Discussions within HHCC and LSHTM participants often conflated decolonisation with concepts like equitable partnerships, Equity, Diversity, and Inclusion (EDI), and localisation.” It is an important point that is referenced throughout the article, but little to no quotations are offered to support it. | Thank you for this. We agree that this is an opportunity to include a powerful quote from one of the study participants. We have added a quote on p. 14: “HHCC member described their understanding, *“Every institution now has an anti-racist action plan, EDI policy. So, for the next couple of years, and beyond, all of these organisations are going to be able to say, Oh, look, we're doing the work, let me show you our policy, let me show you our guideline, we just need more time to implement… Some [organisations] will even call their work decolonial, but do they challenge power, at all?”.”* |
| In the ‘Funding’ section, starting on page 18, is there any perspective from the donors interviewed worth sharing? It would be valuable to incorporate the perspective of their own role in funding structures and decolonization. | Thank you. On p. 21, we have now added further detail: “. Donors highlighted promising examples, such as feminist funds that have begun to shift their funding models towards channelling resources directly to partners in the Global South. A donor shared *“We've been supporting different things. One of them is just getting at the root of what we call southern epistemologies, sort of different ways of knowing. We support an international network of Indigenous women who have what they call their intercultural sort of ways of understanding*”. Another donor described supporting data equity framework, which guides research teams through equity-oriented reflection at each stage of the research process.” |
| **Discussion** | |
| The authors provide several recommendations and suggestions throughout the discussion. Would they consider synthesizing these in a table? This could help highlight key takeaways and provide a clearer guide for those engaging in humanitarian health research and decolonization. | Thank you. We developed a table of key recommendations based on the study findings on p. 31-32 |
| References  - Please ensure the DOI and/or volume and page numbers are available for all Journal Article references | Thank you for the note. We have updated all the references. |
| **Reviewer 2** | |
| Reviewer #2: Overall, this is a very well written, coherent, and meaningful piece of research. I recommend accepting this manuscript for publication, and I have only minor comments and suggestions for the authors to consider. I look forward to seeing this published and citing it in future work. Kudos to the authors for their high level of commitment and integrity, both to decolonising these fields and to the research process - the data speaks for itself. | Thank you so much for the kind feedback and encouragement. |
| Introduction: All of the important concepts are laid out in this section. However, this study focuses on “the humanitarian health sector specifically” (p.8). I am thus wondering if you need to refer to global health (or DGH) at all in your introduction, or if it makes more sense conceptually to dive straight into the sub-section on ‘Colonial Legacies in the Humanitarian Sector’ (and bring in the paragraph starting "Aligning with Aloudat's..." here instead), then ‘Decolonisation Efforts at LSHTM’, then ‘Gaps and Divergent Conceptions…’. I defer to the authors expertise and overarching views of this topic(s) and field(s). Otherwise, for readers like myself who work predominantly in global health and not humanitarian health, perhaps providing a conceptual link between global and humanitarian health (eg, similar and different colonial impacts, legacies, patterns, etc) in the introduction would suffice. | Thank you for the comment. We received a similar comment from the first reviewer on the interchangeable use of global health and humanitarian health and we decided to follow your suggestion and delete the section on decolonizing global health, and replaced that by a brief introduction on page 4 on decolonizing global health, followed by the definition by Aloudat’s, ‘Decolonisation Efforts at LSHTM’, then ‘Gaps and Divergent Conceptions, as you suggested. Thank you for this constructive feedback. |
| **Methods** | |
| Methods:  Only minor feedback. Overall, this section is written clearly.  1. When explaining the Study Design (p.9), I noticed there is no information on participant selection. Would you consider elaborating how participants for the interviews and FGDs were selected ie, were external stakeholders informed by the scoping review? Was the selection purposive? Was maximum variation sampling used? Or snowballing? Were there dependencies between the research team and participants? Were interview participants different or the same as the FGD participants? One sentence on the approach would help to clarify. | Thank you for the sampling and recruitment. On p. 9 and 10, we have added “The study team disseminated a call for participation through HHCC newsletters and LSHTM mailing lists. HHCC members who expressed interest were purposively sampled to ensure diverse perspectives across career stages, and roles. External stakeholders were similarly purposively selected from an existing partner database, taking into consideration diversity in institutional affiliations, geographic locations, and positionalities relevant to HHCC’s research, teaching, and partnerships.”  For the dependency, we added to the ethics section on p. 10: “The research team and participants interacted in a collaborative manner, but no formal dependencies or hierarchical relationships influenced the data collection process.”  On the overlap between interviews and FGD participants, we also added on p. 10: “Four HHCC members participated in both the interviews and FGDs.”  For the approach, we added on p. 11. |
| 2. In Table 1 (p.9), the formatting makes it look as if 'White' is duplicated and there’s no data for ‘Mixed Asian’. I’m also curious as to whether participants had the opportunity to self-define their racial / ethnic backgrounds, or were these categories assigned by the research team? A footnote to the Table might be helpful here. | Thank you so spotting these points. We have amended the formatting and added the missing data for ‘Mixed Asian’ on p. 9.  All the participants self-defined their racial/ethnic background, occupation, grade of seniority and gender. We added this to the paper on p. 9 and changed the table heading to “Self-reported participants racial/ethnic backgrounds” |
| 3. When explaining the FGD methodology (p.10), only the first and second FGDs are described. Was the participatory charter design workshop considered the third FGD? Clarification of the third FGD would help to finalize this paragraph. | Indeed, that’s exactly the case. We changed the description to the following on p. 10: “Subsequently, the third FGD took the form of a participatory charter design workshop to ensure member perspectives informed the development of the charter. |
| 4. When explaining Data Collection & Analysis (p.10), I’d recommend using consistent terminology and not confusing codes with themes ie, “This process involved the development of a codebook informed by the literature review, with additional key CODES…”. I would also avoid saying “we intended to extract race-related THEMES” as themes are not strictly extracted from data, but interpreted or synthesised from the codes. You might consider replacing ‘extract' with 'develop’. As a side-note, is there a need for this sentence? It reads a bit odd that you would mention which themes you did NOT develop, but not state which themes you DID develop — for the authors consideration. | Thanks for spotting this.  We have made changes for consistent and accurate use of terms on p. 11: “The qualitative study was informed by participatory elements, prioritising the lived experiences and knowledge systems of participants, and seeking to critically examine how colonial histories and power structures shape the decolonisation of research, teaching, and partnerships [37]. Data were generated through interviews conducted online using Zoom, with 17 held in English and 3 in Arabic. All interviews were audio-recorded, and those in Arabic were translated into English to ensure consistency in the analysis. Following transcription by SH, the transcripts were inductively and deductively coded using MAXQDA software. Inductive coding involved a close reading of the data to identify patterns, meanings, and concepts emerging directly from participants' accounts. This process involved line-by-line coding, constant comparison, and iterative refinement of codes. Deductive coding was informed by sensitising concepts derived from the scoping review and the overarching research questions. Themes were subsequently developed through a process of synthesis and interpretation of the coded data by SH, with regular discussions among the research team to validate findings. While we intended to develop race-related themes, as suggested in the literature [2, 14], participants ultimately did not focus extensively on this.”  We want to highlight that the literature emphasis on the centrality of race as an analytical category, and we thought we needed to comment on this in the methods, and later in the discussion section, to justify why this paper didn’t use this analysis category- a limitation of this study. |
| 5. The positionality paragraph (p.11) is well received, I appreciate the authors thoughtfulness and diligence in making these statements. | Thank you for the positive feedback and recognition. |
| **Results** | |
| Results: Brilliant results - very meaningful, and communicated with great clarity and transparency. Only minor feedback. | Thank you so much for the encouraging and constructive feedback. |
| 1. I think the second and third themes are well named in that they summarize the main content of the subsequent paragraphs. But the first theme is a bit ambiguous ie, ‘Definitions and institutional readiness’ - of what? Would the authors consider a more specific phrasing for the first theme, something like ‘Defining, understanding, and driving decolonisation efforts institutionally’ (just an example). I think the first theme would also benefit from sub-headings, as done in the second theme. | Thank you for the suggestion. We renamed the first theme “Definitions, understanding and institutional readiness for decolonising the work of HHCC and LSHTM” on p. 13.  We also added sub-headings on p. 13, and P. 15. |
| 2. I’m curious about how the authors decided to attribute some quotes to interviewees ‘of colour’ or ‘white’, and not others. For example, the quote at the top of p.13 does not state the racial / ethnic background of the interviewee whereas many on p.12 do. I am not recommending the authors commit to one or the other, rather find some way of being consistent, perhaps where or if there are sharp contrasts. (My own opinion, if helpful, is that it is interesting to declare for all quotes. This could be done using participant codes after each quote.). | Thank you for your insightful feedback. We appreciate your suggestion regarding the attribution of racial or ethnic backgrounds in our quotes. Our decision to attribute some quotes by racial/ethnic background was done when relevant and when this identification, coupled with the quote/position, would not reveal the identity of the participant. We intended to follow the recommended approach from the literature by centering race as an analytical category and highlight perspectives of racialised minorities, whenever possible. However, we recognise that our analysis did not engage with race in as much depth as initially anticipated, and we were unable to use race as an analytical category and resorted to focusing on the perspectives of racialised minorities. We have expanded on this limitation in the methods and discussion sections to provide further clarity. |
| 3. The paragraph starting “Partners…” (p.15) has language that would be more fitting in the Discussion ie, “This optimism LIKELY…” rather than “This optimism stems from…”. Same later on, “These positive experiences MAY HAVE…”, and “INTERESTINGLY, some HHCC partners…”. Would suggest removing words that imply the authors’ own interpretations, as I think the data supports these statements. | Thank you for flagging this. We have changed the language as per your suggestions on p. 16. |
| 4. Noticed grammar and punctuation potentially missing from some quotes ie, “The historical [insert comma] the colonial history of LSHTM…” (p.16) and “… who have decided. [Capitalise] that we are the…” (p.17). Perhaps check all quotes. | Thank you for spotting these. We have checked all the quotes and made minor changes in grammar and punctuation on p. P.17 and P.18. |
| 5. The sentence starting “One HCC member shared the increasing internal attention…” (p.23), do you have a supporting quote for this? There’s a lot said and by just one interviewee, I think a quote would help illustrate. | Thank you. We added a quote on p. 25: “I think attempts have been made to ensure more equitable representation. And certainly [in] some of the projects that I'm thinking of. There was a recognition that findings and learnings needed to be communicated first and foremost, to the crisis affected communities”. |
| **Discussion** | |
| Discussion: Every point made in the discussion is valuable. However, currently, the discussion flows from conceptualisation to accountability to funding structures to leadership and advocacy to co-planning/-production to partnership dynamics to opportunities to teaching, staff diversity, supervision, and curriculum. The Charter is introduced at different points, and recommendations are woven in here and there. Would the authors consider looking at the structure of the discussion? Some suggestions below. | Thank you so much for the recommendations. Very thoughtful and constructive. We addressed these as mentioned below. |
| 1. As a key study output, the Charter is very interesting and sounds like a great resource. Could this be introduced earlier in the discussion with a panel or box that contains more details ie, its purpose or mandate, goal or aims, objectives, implementation, monitoring? Text on the Charter (p.28, 29, 32) could be removed from the narrative and inserted into this panel / box. | Thank you. We added a box on p. 30 to summarise the purpose, goal and structure of the charter and implementation guidance. |
| 2. Could the authors also consider a separate panel or box for key recommendations from this study, or ‘good practices’ as mentioned in the Abstract? I noticed that recommendations are interspersed (p. 28, 29, 30, 31, 32, 33) when they could be consolidated in a panel / box, perhaps merged with the Charter. Similarly then, text on recommendations could be removed from the narrative. | Thank you we developed a table of key recommendations based on the study findings on p. 36-37. |
| 3. The authors mention “all stages of the research process” (p.29). What are these stages, and do the main barriers articulated in the discussion map to these stages? This might be one way to bring a bit more structure to this section, starting with the first stage in this process. | Thank you. We have clarified the stages in the text, and introduced minor changes to make it clearer on p. 32. |
| 4. The paragraph starting “Unlike institutions…” (p.34) is partially repeated halfway down the next page. | Thank you for spotting this. We deleted this paragraph in P.34 |
